# Psychosocial and mental health outcomes associated with gender-affirming interventions among transgender youth: a scoping review

**DOI:** 10.3389/fpubh.2026.1910466

**Published:** 2026-09-16

**Authors:** Balaji Murugesan, Visalakshmi S, Venkataraman Prabhu, Vijayalakshmi Chellappa, Remya Lathabhavan, Bhavik M. Panchasara, Siva Vijayakumar Tharumasivam, Srividhya Vasudevan, Saranya Sundarraju, Ponnuchamy Lingam

**Affiliations:** 1 Division of Medical Research, SRM Medical College and Hospital SRMIST, Kattankulathur, India; 2 Department of Management, Central University of Tamil Nadu, Tiruvarur, India; 3 Department of Statistics and Applied Mathematics, Central University of Tamil Nadu, Tiruvarur, India; 4 Indian Institute of Management, Bodh Gaya, Bihar, India; 5 Saurashtra University, Rajkot, Gujarat, India; 6 Department of Biotechnology, Dhanalakshmi Srinivasan University, Trichy, Tamil Nadu, India; 7 Independent Researcher at Tamil Nadu, Chennai, India; 8 Department of Psychiatric Social Work, National Institute of Mental Health and Neurosciences (NIMHANS), Bengaluru, India

**Keywords:** adolescents, gender-affirming care, mental health, psychosocial outcomes, scoping review, transgender health, transgender youth

## Abstract

**Introduction:**

Transgender youth are a highly vulnerabilized population, facing stigma, discrimination, family rejection, and social exclusion that elevate their risk of depression, anxiety, and suicidal ideation. Gender-affirming interventions-medical, psychological, social, and structural-have been proposed to improve health and wellbeing in this group, but evidence on their psychosocial and mental health impact remains scattered across heterogeneous contexts and intervention types. This scoping review aimed to map and synthesize available evidence on psychosocial and mental health outcomes associated with gender-affirming interventions among transgender youth.

**Methods:**

Following JBI methodology and the PRISMA-ScR checklist, we searched PubMed, Scopus, Embase, Web of Science, and CINAHL in August 2025 for peer-reviewed, English-language articles published between January 2011 and July 2025. Eligible studies addressed gender-affirming interventions or experiences among transgender and gender-diverse individuals and reported psychosocial or mental health outcomes.

**Results:**

Of 1,207 records identified, 17 studies met eligibility criteria, comprising 123,428 participants (range: 15–120,617 per study). Designs included prospective cohort (*n* = 7), cross-sectional (*n* = 7), retrospective (*n* = 2), and qualitative (*n* = 1) studies, primarily from the US, UK, Australia, Sweden, Italy, and India. Evidence consistently linked gender-affirming interventions to improvements in quality of life, psychological wellbeing, emotional functioning, and social participation. Medical interventions (puberty suppression, hormone therapy, surgery) were generally associated with reduced depression, anxiety, distress, and suicidality, though one study found persistently elevated suicide risk decades post-surgery. Non-medical interventions-psychotherapy, family support, social transition, and community-based support-also improved mental health. Conversely, stigma, discrimination, family rejection, and structural healthcare barriers were consistently linked to poorer outcomes, particularly in studies from India.

**Conclusion:**

Gender-affirming interventions are associated with improved psychosocial and mental health outcomes among transgender youth, but optimal wellbeing depends on more than medical intervention alone, requiring family support, inclusive education, affirmative healthcare systems, and supportive policy. Substantial gaps remain for non-Western, low- and middle-income, and non-binary/gender-diverse populations, highlighting the need for research testing whether existing frameworks translate across sociocultural contexts.

## 1 Introduction

A proper understanding of transgender identities is crucial to creating an inclusive society, promoting social justice, and promoting health and wellness among all people. While there has been a considerable rise in awareness of transgender identities among different groups of stakeholders (academicians, policymakers, and the general public), the reality of the situation is that transgender youths are still facing many problems in terms of healthcare, education, and employment (1, 2). These issues have to be addressed using effective policies, adequate access to gender-affirming care, and a supportive environment.

Transgender youths constitute one of the most vulnerabilized subpopulations around the world, as a result of social stigma and structural barriers. The factors that negatively affect their overall wellness include stigma and discrimination, rejection by the family, bullying, and social isolation. These elements play a considerable role in the development of psychological issues (including depression and anxiety), self-harm, low self-esteem, and poor quality of life (3).

There is evidence from research in multiple countries of a rising number of adolescents identifying as transgender and/or gender-diverse, though prevalence estimates vary considerably depending on survey methodology, age range, and cultural context. In the United States, epidemiological studies estimate that 0.7% of adolescents between ages 13 and 17 identify as transgender (4). Comparable adolescent population surveys from other regions report similar or higher figures: a nationally representative high school survey in New Zealand found that 1.2% of students identified as transgender (5), while a large Finnish adolescent survey reported 0.6% opposite-sex identification and 3.2% non-binary or other gender identity (6). In Brazil, the first nationally representative adult prevalence estimates for Latin America found that transgender individuals comprised 0.69% and non-binary individuals 1.19% of the sampled population, with no significant variation across the country's five geographic regions (7) comparable adolescent-specific prevalence data for Latin America remain unavailable. By contrast, robust population-level prevalence data are largely absent for most of Asia, Africa, and the Middle East, reflecting differences in legal gender recognition, survey infrastructure, and research investment across regions, rather than differences in the underlying prevalence of transgender identity. These disparities in epidemiological visibility do not necessarily reflect the true distribution of transgender youth across different settings but instead point to geographical and contextual differences in legal recognition, healthcare system structure, cultural attitudes toward gender diversity, and research funding priorities. These factors likely shape the psychosocial experiences of transgender adolescents in ways that remain comparatively understudied in regions beyond North America, Western Europe, and Australia.

Considering the multidimensional nature of the determinants of health and the wellbeing of transgender individuals, there has been growing interest in the development of interventions to improve their situation. For example, gender-affirming interventions such as social transition, hormone therapy, surgeries, and other services related to psychological wellbeing have shown benefits for mental health, psychological wellbeing, and quality of life of transgender youth (8, 9).

Although there has been significant literature development on this issue, current evidence still appears scattered due to the heterogeneity of interventions being considered, target populations, and geography of the study samples used. Indeed, much of the available literature comes from high-income nations, with inadequate contribution from low- and middle-income regions and from transgender people who exist within non-binary gender categories. This concentration is not a neutral gap in coverage: it means that the theoretical models most commonly used to interpret transgender youth mental health, including the Gender Affirmation Model and the Minority Stress Model, have themselves been developed and validated primarily using data from Western, English-speaking populations, raising the question of whether their constructs (e.g., what counts as “family support” or “social affirmation”) translate directly to sociocultural contexts with different family structures, community systems, and legal environments. Furthermore, the underrepresentation of non-binary and gender-diverse populations within existing research risks conflating the experiences of binary transgender individuals with a more heterogeneous population whose relationships to medical and social gender affirmation may differ substantially. Moreover, to the best of our knowledge, no recent scoping review exists to summarize the psychosocial and mental health effects associated with gender-affirming interventions in transgender youth across this heterogeneous evidence base. It is therefore important to map the existing evidence, not only to identify empirical gaps for future primary research, but also to critically examine whether existing theoretical frameworks require adaptation or extension before being applied to underrepresented populations and contexts.

Therefore, this scoping review aimed to systematically map and synthesize the evidence regarding psychosocial and mental health outcomes associated with gender-affirming interventions among transgender youth. Specifically, the review sought to characterize quality of life, psychosocial stressors, structural influences, mental health outcomes, and supportive interventions related to wellbeing in this population. By synthesizing the available evidence across the settings represented in the literature, this review aims to inform the development of evidence-based interventions that promote mental wellbeing and social inclusion for transgender youth.

## 2 Methods

### 2.1 Design

This scoping review method and reporting were based on the Joanna Briggs Institute (JBI) methodology for scoping reviews (10, 11) and the Preferred Reporting Items for Systematic Reviews and Meta-Analyses extension for Scoping Reviews (PRISMA-ScR) guidelines (12). Unlike Systematic reviews, which address a relatively narrow range of quality-assessed studies, systematic scoping reviews enable dealing with broader questions, mapping the key concepts underpinning a research area and the main sources and types of evidence with a range of methodologies, and do not require quality assessment.

### 2.2 Search strategy

The extensive literature search included studies published between January 2011 and July 2025, identified across PubMed, Scopus, Cumulative Index to Nursing and Allied Health Literature, Embase, and Web of Science databases. The January 2011 start date was selected because this period broadly coincides with the emergence of more standardized, affirmation-based clinical and research approaches to transgender youth healthcare internationally, including the World Professional Association for Transgender Health (WPATH) Standards of Care, Version 7 (13), which preceded the current Version 8 (14) and shaped clinical and research practice throughout the review period. This starting point allowed the review to capture the body of evidence generated under a broadly consistent clinical and conceptual framework for gender-affirming care, rather than an arbitrarily selected date. The final search across all databases was performed in August 2025. The search strategy began with a preliminary limited search of MEDLINE to identify relevant terms and keywords. The search included terms and keywords related to the two main concepts of this review: gender-affirming care and psychosocial or mental health, combined using Boolean operators (AND/OR). Specifically, the keywords and terms included:

#### 2.2.1 Gender-affirming care

Terms such as “gender-affirming care,” “gender-affirming intervention,” “hormone therapy,” “puberty suppression,” “social transition,” “psychotherapy”.

#### 2.2.2 Psychosocial or mental health

Terms such as “mental health,” “psychological wellbeing,” “depression,” “anxiety,” “suicidality,” “quality of life,” and “psychosocial outcomes”.

Search terms were adapted to the indexing requirements of each database. Searches were restricted to peer-reviewed articles published in English. This restriction reflects practical resource constraints within the review team, namely the absence of reliable in-house translation capacity for the accurate extraction of nuanced psychosocial and clinical outcome data from non-English sources. While machine translation tools are increasingly available, we did not consider them sufficiently reliable for this review, given the risk of misrepresenting culturally specific psychological constructs, colloquial expressions of distress, and validated instrument items whose meaning may not translate directly across languages. We acknowledge that this decision privileges English-language, and by extension often Western-affiliated, research output, and may have excluded relevant evidence generated in non-English-speaking contexts, including regions such as Latin America, East Asia, and parts of continental Europe where research on transgender youth mental health is emerging. We address this constraint explicitly as a limitation of the evidence base in Section 4.1. Animal studies were also excluded. To ensure comprehensive coverage, the reference lists of all included studies were manually screened to identify additional eligible articles not captured through database searches.

### 2.3 Eligibility criteria

This scoping review utilized the JBI Population, Concept, Context framework for guidance (10) (Table 1). The population included transgender and gender-diverse individuals in adolescence and adulthood. For the purposes of this review, “gender-diverse” refers to individuals whose gender identity or expression differs from the sex assigned to them at birth and does not conform to binary categories of male or female, including but not limited to nonbinary, genderqueer, agender, and culturally specific gender identities such as Hijra. The concept involved gender-affirming interventions, encompassing medical, psychological, social, and structural affirmation, as well as mental health indicators that are related to access to, or lack of access to, gender-affirming interventions. As the context is unrestricted to geographical location, type of healthcare system, or sociocultural context, a wide array of evidence on gender affirmation and mental health can be captured.

**Table 1:** Inclusion and exclusion criteria based on the Joanna Briggs Institute (JBI) Population–Concept–Context (PCC) framework.

PCC framework	Inclusion criteria	Exclusion criteria
**Population**	Transgender and gender-diverse individuals who received or experienced gender-affirming interventions	Studies involving only cisgender populations or studies that did not report separate findings for transgender or gender-diverse individuals
**Concept**	Gender-affirming interventions, including medical (puberty suppression, hormone therapy, surgery), psychological, social, and structural forms of affirmation. Studies reporting psychosocial or mental health outcomes such as depression, anxiety, psychological distress, suicidality, quality of life, wellbeing, stress, and social functioning	Studies not addressing gender-affirming interventions or experiences of gender affirmation. Studies not reporting psychosocial or mental health outcomes
**Context**	Any geographical location, healthcare setting, educational setting, community setting, or sociocultural context. No restrictions on country or healthcare system	No specific exclusions based on context
**Study**	Peer-reviewed quantitative, qualitative, and mixed-methods studies published in English between January 2011 and July 2025	Reviews, systematic reviews, scoping reviews, editorials, commentaries, conference abstracts, study protocols, non-English publications, studies with inaccessible full texts, and studies lacking relevant outcome data

Eligible studies had to be peer-reviewed publications written in the English language (rationale detailed in Section 2.2). All types of quantitative, qualitative, and mixed methods research that explore the topic of gender-affirming interventions or experiences of gender affirmation and provide information on psychosocial and mental health outcomes were eligible. The list of relevant mental health outcomes includes, but is not limited to, depression, anxiety, psychological distress, stress, suicidality, quality of life, and general wellbeing. Since the aim of the current scoping review is the exploration of the available evidence base, all methodological approaches are acceptable.

Non-original studies, such as reviews, editorials, commentaries, conference abstracts, and study protocols, were also not included. In addition, studies that addressed only cisgender populations and failed to report separate results for transgender or gender diverse individuals were not included. Furthermore, studies failing to measure relevant outcomes related to mental health, having inaccessible full-text papers, or not written in English were also excluded from the review.

The inclusion criteria specified that only peer-reviewed literature would be considered for synthesis, in order to maintain a feasible and consistent scope for evidence mapping within this review. While relevant data could potentially also be found in reports, organizational publications, policy briefs, and other gray literature, the heterogeneity in format, structure, and reporting conventions across such sources posed challenges for consistent data charting and synthesis within the thematic domains used in this review. Limiting inclusion to peer-reviewed literature allowed for a more systematic and comparable mapping of evidence surrounding gender-affirming interventions and mental health outcomes in transgender and gender diverse individuals, consistent with the scoping review's aim of charting the available evidence base in a structured and reproducible manner.

No geographic or setting limits were set to facilitate obtaining an overview of the current state of evidence regarding the relationship between gender affirming interventions and mental health outcomes in transgender and gender diverse populations.

### 2.4 Data extraction

The data extraction was done based on a standard framework for data charting that was consistent with the goals of the current review. Data extracted included: year of publication and country, research design, number of participants, description of participants, gender affirmation therapy intervention/exposure, mental health and psychosocial outcomes measured, instruments used, and results obtained.

### 2.5 Analysis and reporting

As there is diversity among the included studies with regard to design, demographics, types of gender-affirming interventions, and outcomes, a narrative approach to synthesizing the findings was taken. To achieve the objectives of this review, a qualitative content analysis was conducted in line with the recommendations of Pollock et al. (11) for the extraction, analysis, and presentation of results in scoping reviews. Initially, two investigators independently examined the objectives, key concepts, and reported outcomes of all included articles. Based on this examination, the studies were categorized according to their type of gender-affirming intervention, age range, and location, and were subsequently clustered into five thematic categories aligned with the review objectives: Quality of Life, Psychosocial Stress and Minority Stress, Mental Health Outcomes Following Medical Interventions, Non-medical Psychotherapeutic Interventions, and Structural and Policy Determinants. The results of each category are presented as a narrative descriptive summary accompanied by tabulated results (Table 2), as appropriate.

**Table 2:** Thematic synthesis of psychosocial and mental health outcomes across included studies.

Theme	Included studies	Main findings
Quality of life	Pelusi et al., (15) Poguri et al., (17) Foster Skewis et al., (22) Majumder et al., (26) Lindqvist et al. (29)	Gender-affirming interventions improved quality of life, life satisfaction, emotional well-being, and social functioning
Psychosocial stress and minority stress	Strauss et al., (9) Sartaj et al., (18) Reisner et al., (24) Toomey et al., (25) Sevelius et al. (27)	Stigma, discrimination, family rejection, and social exclusion negatively affected mental health outcomes
Mental health outcomes following medical interventions	Tucker et al., (16) Biggs et al., (20) Tordoff et al., (21) Dhejne et al., (28) Olson-Kennedy J et al. (30)	Medical interventions were generally associated with reduced depression, anxiety, and suicidality, although some long-term findings were mixed
Non-medical psychotherapeutic interventions	Kuper et al., (19) Costa et al. (23)	Gender-affirming psychotherapy and psychological support improved well-being and reduced depressive symptoms
Structural and policy determinants	Poguri et al., (17) Sartaj et al., (18) Majumder et al., (26)	Access to affirming healthcare, supportive policies, and legal protections influenced psychosocial outcomes

## 3 Results

The database search identified 1,207 records. After removal of duplicates (*n* = 69), 1,138 records underwent title and abstract screening, resulting in the exclusion of 1,028 records based on predefined eligibility criteria. Of the 110 reports sought for retrieval, 5 could not be retrieved (3 due to inaccessible full text, 2 due to non-English publication), leaving 105 full-text reports assessed for eligibility. Of these, 88 were excluded for failing to meet the predefined inclusion criteria, including review articles (*n* = 7), studies not meeting inclusion criteria (*n* = 36), and studies that did not assess relevant outcomes (*n* = 45), resulting in 17 studies meeting the inclusion criteria for the final scoping review (Figure 1) (9, 15–30).

**Figure 1:**
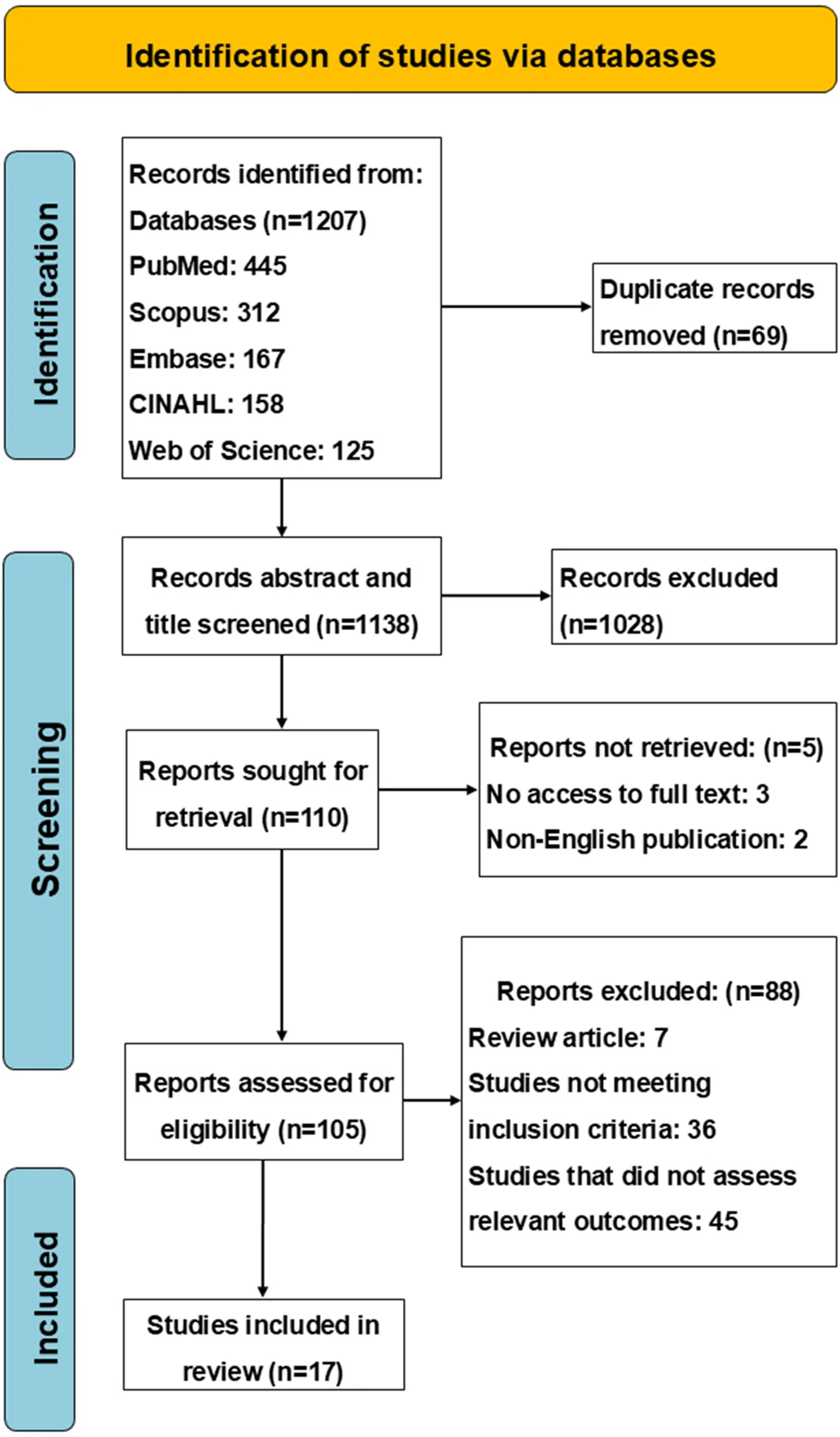
Flow chart diagram of the study selection process based on PRISMA-ScR (12).

### 3.1 Characteristics of included studies

The included studies represented diverse geographical settings, with most originating from the United States (16, 19, 21, 24, 25, 27, 30), United Kingdom (20, 23), Australia (9, 22), Sweden (28, 29), Italy (15), and India (17, 18, 26). Sample sizes ranged from 15 participants (17) to 120,617 participants (25), reflecting substantial methodological heterogeneity, with a combined total of 123,428 participants across the 17 included studies. Study designs included 7 prospective cohort studies (15, 19, 21–23, 29, 30), 7 cross-sectional studies (9, 16–18, 24–26), 2 retrospective studies (20, 28), and 1 Qualitative study (27).

Interventions and exposures encompassed a broad range of gender-affirming approaches, including medical interventions such as puberty suppression using gonadotropin-releasing hormone (GnRH) agonists (21, 23, 30), gender-affirming hormone therapy (15, 16, 22), and surgical procedures (16, 28, 29); non-medical psychosocial interventions such as psychotherapy, family support, social transition, and community-based support (19, 22); and structural or policy-level factors addressing healthcare access, legal protections, stigma, and discrimination (18, 24, 26, 27). Reported outcomes primarily focused on mental-health and psychosocial indicators, including depression, anxiety, perceived stress, suicidality, self-harm, quality of life, psychological wellbeing, body image, and health-risk behaviors (9, 20, 21, 27, 29). The detailed characteristics of included studies are presented in (Supplementary material 1).

### 3.2 Quality of life

Quality of life (QoL) outcomes were assessed in several studies using standardized instruments such as the World Health Organization Quality of Life—BREF (WHOQOL-BREF) and Short Form Health Survey−36 (SF-36). Studies from India consistently reported poor overall QoL among transgender individuals, with the social and environmental domains being the most adversely affected due to stigma, discrimination, social exclusion, and limited family support. One study reported extremely low social QoL scores, alongside universal depressive symptoms and high rates of suicidality among transfeminine individuals (17). Similarly, another study established a link between family acceptance and support and positive QoL outcomes, while discrimination was linked with poorer wellbeing (26).

Research findings on gender-affirming therapies also indicated improvements in QoL with their use. For instance, one study recorded improvement in life satisfaction 1 year after testosterone treatment in transmen (15). Similar trends were reported in another study, which found improvement in a number of QoL dimensions 1 year after gender-affirming surgery, although these improvements reached a plateau after that period (29). A prospective analysis conducted in a further study showed improvement in emotional wellbeing and social function with masculinizing hormone treatment, while QoL improvements were less evident for feminizing hormone therapy (22).

### 3.3 Psychosocial stress and minority stress

Transgender youth experienced substantial psychosocial stress arising from stigma, discrimination, family rejection, trauma, social exclusion, and barriers to accessing affirming healthcare. One study reported high levels of psychological distress among transgender and gender-diverse youth, with family rejection, homelessness, and adverse life events identified as significant predictors of poor mental health outcomes (9). Indian studies further highlighted the impact of childhood sexual abuse, economic marginalization, social exclusion, and community-level stigma on psychosocial wellbeing (18).

Minority stress was further reinforced by negative societal and institutional attitudes toward transgender individuals. Another study identified discrimination and barriers to healthcare access as major predictors of depression, anxiety, and poor mental health outcomes among transgender adults (24). In contrast, affirming healthcare interactions and supportive clinical environments were associated with reduced psychological distress and lower suicide risk (9).

Several studies also documented elevated rates of health-risk behaviors, particularly self-harm and suicide attempts. One study reported lifetime self-harm among 79% of transgender and gender-diverse youth and suicide attempts among 48% of participants (9). National survey data from another study demonstrated that suicide attempt prevalence was substantially higher among transgender and nonbinary youth than among their cisgender peers (25). Qualitative findings from further study suggested that unsafe body modification practices and sexual risk behaviors may function as coping mechanisms in response to chronic stigma and unmet gender affirmation needs (27). Despite the presence of community-based support systems such as Hijra community networks, which offered psychological strength and resilience, these alone were often insufficient to entirely ameliorate the consequences of minority stress and structural discrimination (18).

### 3.4 Post-medical intervention mental health outcomes

The literature on the effects of gender-affirmation medical treatments on mental health tended to be positive, especially over the short and medium term. One study found that adolescents receiving either puberty-blocking treatments or gender-affirming hormone therapy showed decreased depression and suicidality after 12 months (21). Supporting the pharmacological basis of these interventions, a retrospective study demonstrated that both Vantas (50 mcg/day) and Supprelin LA (65 mcg/day) histrelin implants effectively suppressed the hypothalamic–pituitary–gonadal axis in early-to-mid pubertal transgender youth, with comparable reductions in gonadotropin and sex steroid levels between the two formulations, indicating that both are clinically effective options for pubertal suppression (30). Likewise, another study found that suicide risk was lower among transgender veterans using hormone therapy and/or undergoing gender-affirming surgeries (16).

However, findings were not entirely consistent across studies. One study reported no significant psychological benefit following puberty suppression in a small cohort from the United Kingdom and noted a possible increase in self-harm behaviors, highlighting the need for cautious interpretation and longer-term evaluation (20). Furthermore, long-term registry data from Sweden reported in another study indicated persistently elevated rates of psychiatric morbidity and suicide risk among individuals who had undergone gender-affirming surgery, emphasizing the importance of ongoing mental health support and long-term follow-up rather than viewing medical transition as a standalone intervention (28).

### 3.5 Non-medical psychotherapeutic interventions

Non-medical, gender-affirming psychotherapeutic interventions demonstrated beneficial effects on mental health and psychosocial functioning. One study reported significant reductions in depressive symptoms and improvements in overall wellbeing following a 12-week gender-affirming cognitive behavioral therapy program for transgender and nonbinary adolescents (19). In line with these findings, another study concluded that psychological interventions alone yielded only minor benefits regarding improvement in global functioning, with more positive effects when such interventions are used alongside puberty suppression (23).

Thus, the availability of affirming and helpful psychological counseling among other forms of psychosocial support, is an important factor that warrants attention when working with transgender individuals. Mental health interventions proved beneficial regardless of an individual's decision to pursue medical transition.

### 3.6 Determinants on a structural and policy level

Structural and policy-level determinants had a significant impact on the psychological wellbeing, QoL, and health status of transgender individuals. In the examined studies, lack of proper legal protection, inadequate access to healthcare facilities, socio-economic difficulties, and structural discrimination were associated with poorer health-related outcomes (17, 18, 21). These factors are especially pertinent to low and middle-income countries where access to services and health systems remain relatively limited.

In particular, the World Professional Association for Transgender Health WPATH Standards of Care highlights the importance of timely access to personalized, multidisciplinary care, mental health interventions, and family and community support, as well as the use of evidence-based gender-affirming approaches (14).

## 4 Discussion

This scoping review synthesized evidence from 17 studies (9, 15–30) examining psychosocial and mental health outcomes associated with gender-affirming interventions among transgender youth across diverse geographical settings. Overall, the findings suggest a consistent association between gender-affirming interventions and improvements in wellbeing, quality of life, and mental health outcomes.

Several studies included in this review reported reductions in depression, anxiety, and suicidality following hormone therapy and puberty suppression (15, 16, 19, 21, 29), a pattern consistent with longitudinal prospective evidence from outside this review's sample (31). This convergence lends further weight to the Gender Affirmation Model (27), which posits that psychological wellbeing improves as gender identity is increasingly recognized and affirmed across social, psychological, and physical domains. However, our review also surfaced findings that complicate this generally positive picture. One study (20) reported no statistically significant improvement in psychological functioning following puberty suppression and noted a possible increase in self-harm within a UK cohort, while another study (28) found persistently elevated long-term suicide risk among adults who had undergone gender-affirming surgery decades earlier. These discrepant findings resist easy reconciliation with a straightforward “affirmation improves outcomes” narrative, and instead suggest that the relationship between medical affirmation and long-term psychiatric risk may be moderated by factors this review's evidence base cannot fully resolve, including cohort era, length of follow-up, comorbid trauma exposure, and the availability of concurrent psychosocial support.

The finding that psychosocial affirmation, including family support, peer relationships, and school inclusion, emerged as being at least as influential as medical affirmation is consistent with the Minority Stress Model (32), which frames transgender mental health disparities as arising primarily from stigma, discrimination, victimization, and social exclusion rather than from gender identity itself. This is corroborated by evidence from outside this review's sample: transgender adults in the United States report significantly poorer general, physical, and mental health than cisgender peers independent of treatment status (33), and experiences of transgender-related discrimination in healthcare, employment, and housing remain widespread, particularly among socioeconomically marginalized individuals (34). Within this review, one study (21) found that quality of life was positively correlated with family and social support, while discrimination predicted poorer wellbeing. Similarly, another study (24) reported that discrimination and barriers to healthcare access were major predictors of depression and poor mental health outcomes among transgender adults. Read together with the WPATH Standards of Care Version 8′s (14) call for multidisciplinary, non-biomedical affirming care, this pattern suggests that psychosocial and structural determinants may function not merely as adjuncts to medical intervention but as independent, and in some cases equally load-bearing, drivers of mental health outcomes. This has a direct implication for how gender-affirming care is conceptualized: framing affirmation solely as a clinical or medical pathway risks under-investing in the family, school, and community-level interventions that this review's evidence suggests may carry comparable weight.

Timing of intervention also appears to matter. Within this review, the same study (21) found that the greatest reductions in depression and suicidality were observed among youth who initiated both puberty blockers and hormone therapy earliest, suggesting that earlier and more comprehensive access to gender-affirming care may confer greater psychosocial benefit than delayed or partial intervention. However, medical treatment alone appears insufficient: counseling, peer-support groups, and trauma-informed, culturally sensitive mental health services continue to play an essential role in comprehensive gender-affirming care for transgender youth, consistent with a broader multidisciplinary model of care (22), and with wider evidence supporting affirmative cognitive-behavioral and school- or community-based interventions for sexual and gender minority youth (38–40).

A further pattern worth foregrounding is the geographic disparity evident across the included studies. Findings from countries with established, publicly or institutionally funded gender-affirming healthcare infrastructure, such as the United States (16, 19, 21, 24, 25, 27, 30), Sweden (28, 29), and the United Kingdom (20, 23), were broadly favorable, while studies from India (17, 18, 26) documented substantially higher baseline psychological distress alongside considerably more limited access to affirming services, reflecting sociocultural stigma, gaps in specialized healthcare training, and inconsistent implementation of existing legal protections. This association between healthcare system infrastructure and favorable outcomes reflects the specific structure, funding, and political status of the systems studied at the time of data collection, rather than a stable or universal relationship with national income level; recent political shifts in several high-income countries, including new restrictions on gender-affirming care for minors, demonstrate that such access is neither static nor guaranteed by a country's economic development status. Notably, this geographic variation in psychological burden is not confined to lower-resourced contexts: a comparison of treatment-seeking adolescents across four specialist gender clinics in high-income European countries similarly found marked cross-national differences in baseline emotional, behavioral, and peer-relationship problems, with adolescents in the UK showing the highest prevalence of difficulties among the clinics studied (35). This suggests that even within well-resourced healthcare systems, national and clinic-level context shapes the psychological profile of transgender youth prior to intervention, a factor that should be accounted for when comparing outcomes across studies and settings. This broader pattern is consistent with earlier calls in the global health literature to interrogate the applicability of Western-derived clinical and theoretical frameworks, including the Gender Affirmation and Minority Stress models themselves, to non-Western sociocultural contexts (36, 37). In India specifically, the Transgender Persons (Protection of Rights) Act, 2019 represents a notable legal milestone, yet implementation gaps, limited clinician expertise in gender-affirming care, and resulting risks of misdiagnosis and stigmatization continue to constrain its practical impact (18, 36). An intersectional lens further complicates this picture: caste, class, and religious hierarchies intersect with gender identity to compound the marginalization experienced by hijra and kothi communities in India, shaping their access to social and economic resources (37). This suggests that the applicability of Western-derived affirmation frameworks may be conditional rather than universal, and that future theoretical and empirical work should more explicitly test whether constructs such as “family support” or “social affirmation” carry equivalent meaning and protective weight across sociocultural contexts.

Taken together, these findings support both the Gender Affirmation Model (27) and the Minority Stress Model (32) as complementary rather than competing explanatory frameworks, while also indicating that neither model, on its own, fully accounts for the discrepant long-term findings or the pronounced geographic disparities observed across this evidence base. This points toward a need for context-sensitive extensions of these frameworks, ones that explicitly incorporate structural determinants such as legal protection, healthcare system maturity, and intersecting social hierarchies, rather than treating gender affirmation as a uniform therapeutic construct. Accordingly, we argue that gender affirmation should be understood not solely as a clinical or therapeutic pathway, but as a matter of public health and human rights advocacy, requiring policy, structural, and community-level intervention alongside clinical care.

### 4.1 Limitations

This scoping review has several limitations that should be considered when interpreting the findings. First, eligibility was restricted to peer-reviewed, English-language publications, which may have excluded relevant evidence published in regional languages or in gray literature such as government reports and policy briefs, particularly from non-English-speaking and low- and middle-income contexts. Second, unpublished studies were not considered, raising the possibility that publication bias has shaped the body of evidence synthesized here, as studies with null or negative findings may be underrepresented in the peer-reviewed literature. Third, the substantial heterogeneity across included studies in design, population characteristics, intervention type, and outcome measures limits the extent to which findings can be directly compared or generalized across settings; consequently, no causal relationships between gender-affirming interventions and psychosocial outcomes can be inferred from this synthesis. Finally, the majority of included studies employed observational or cross-sectional designs, which are inherently susceptible to reporting bias and cannot establish temporal or causal relationships between exposures and outcomes.

## 5 Conclusion

In conclusion, this scoping review has synthesized the available published evidence on the psychosocial and mental health outcomes of gender-affirming interventions among transgender youth. In the 17 included articles, both medical and non-medical gender-affirming interventions have shown to contribute to improvements in the overall quality of life, psychological wellbeing, and mental health outcomes, including reduction of depression, anxiety, psychological distress, and suicidality among other positive effects. It is also worth mentioning that some positive results are not only due to the presence of medical treatments but depend on family support, social affirmation, inclusive education, and affirmative healthcare facilities as well.

Despite all the potential positive aspects of gender-affirming interventions, some serious obstacles still exist in terms of the health-related problems of transgender youth. Stigma, discrimination, structural inequities, and lack of access to appropriate healthcare are associated with negative impacts on the wellbeing of transgender youth, especially those from lower- and middle-income countries. Thus, it becomes evident that a person-centered approach combining the above-mentioned factors can be an important step toward positive changes in health care.

Future studies should focus on the issues of long-term outcomes, geographical expansion to underrepresented countries, and the population of non-binary/gender-diverse youth.

## Data Availability

The original contributions presented in the study are included in the article/Supplementary material, further inquiries can be directed to the corresponding author.

## References

[1] Herrmann L Bindt C Hohmann S Becker-Hebly I. Social media use and experiences among transgender and gender diverse adolescents. Int J Transgend Health. (2023) 25:36–49. doi: 10.1080/26895269.2023.2252410 38328588 PMC10846420

[2] Hemmige BD Gokula Krishnan B. Media representation of transgenders in India: patterns, perceptions and policy implications. Int J Environ Sci. (2025) 11:3256–69. doi: 10.64252/atppe626

[3] Lal BS. Transgenders community in India: socioeconomic health and psychological conditions. J Res Soc Sci Humanit. (2023) 2:1–8. doi: 10.56397/JRSSH.2023.11.01

[4] Herman JL Flores AR Brown TNT Wilson BDM Conron KJ. Age of Individuals Who Identify as Transgender in the United States. Los Angeles, CA: Williams Institute, UCLA School of Law (2017).

[5] Clark TC Lucassen MF Bullen P Denny SJ Fleming TM Robinson EM . The health and well-being of transgender high school students: results from the New Zealand adolescent health survey (Youth'12). J Adolesc Health. (2014) 55:93–9. doi: 10.1016/j.jadohealth.2013.11.008 24438852

[6] Kaltiala-Heino R Bergman H Työläjärvi M Frisén L. Gender dysphoria in adolescence: current perspectives. Adolesc Health Med Ther. (2018) 9:31–41. doi: 10.2147/AHMT.S135432 29535563 PMC5841333

[7] Spizzirri G Eufrásio R Lima MCP de Carvalho Nunes HR Kreukels BPC Steensma TD . Proportion of people identified as transgender and non-binary gender in Brazil. Sci Rep. (2021) 11:2240. doi: 10.1038/s41598-021-81411-4 33500432 PMC7838397

[8] de Graaf NM Huisman B Cohen-Kettenis PT Twist J Hage K Carmichael P . Psychological Functioning in Non-binary Identifying Adolescents and Adults. J Sex Marital Ther. (2021) 47:773–84. doi: 10.1080/0092623X.2021.1950087 34344272

[9] Strauss P Cook A Winter S Watson V Wright Toussaint D Lin A. Associations between negative life experiences and the mental health of trans and gender diverse young people in Australia: findings from Trans Pathways. Psychol Med. (2020) 50:808–17. doi: 10.1017/S0033291719000643 31280740

[10] Peters MDJ Godfrey CM McInerney P Soares CB Khalil H Parker D. Methodology for JBI scoping reviews. In: The Joanna Briggs Institute Reviewers' Manual 2015. Adelaide (AU): The Joanna Briggs Institute (2015). 1–24.

[11] Pollock D Peters MDJ Khalil H McInerney P Alexander L Tricco AC . Recommendations for the extraction, analysis, and presentation of results in scoping reviews. JBI Evid Synth. (2023) 21:520–32. doi: 10.11124/JBIES-22-00123 36081365

[12] Tricco AC Lillie E Zarin W O'Brien KK Colquhoun H Levac D . PRISMA Extension for scoping reviews (PRISMA-ScR): checklist and explanation. Ann Intern Med. (2018) 169:467–73. doi: 10.7326/M18-0850 30178033

[13] Coleman E Bockting W Botzer M Cohen-Kettenis P DeCuypere G Feldman J . Standards of care for the health of transsexual, transgender, and gender-nonconforming people, version 7. Int J Transgend. (2011) 13:165–232. doi: 10.1080/15532739.2011.700873

[14] Coleman E Radix AE Bouman WP Brown GR de Vries ALC Deutsch MB . Standards of care for the health of transgender and gender diverse people, version 8. Int J Transgend Health. (2022) 23:S1–S259. doi: 10.1080/26895269.2022.2100644 36238954 PMC9553112

[15] Pelusi C Costantino A Martelli V Lambertini M Bazzocchi A Ponti F . Effects of three different testosterone formulations in female-to-male transsexual persons. J Sex Med. (2014) 11:3002–11. doi: 10.1111/jsm.12698 25250780

[16] Tucker RP Testa RJ Simpson TL Shipherd JC Blosnich JR Lehavot K. Hormone therapy, gender affirmation surgery, and their association with recent suicidal ideation and depression symptoms in transgender veterans. Psychol Med. (2018) 48:2329–36. doi: 10.1017/S0033291717003853 29331161

[17] Poguri M Sarkar S Nambi S. A pilot study to assess emotional distress and quality of life among transgenders in South India. Neuropsychiatry. (2016) 6:22–7. doi: 10.4172/Neuropsychiatry.1000113

[18] Sartaj D Krishnan V Rao R Ambekar A Dhingra N Sharan P. Mental illnesses and related vulnerabilities in the Hijra community: a cross-sectional study from India. Int J Soc Psychiatry. (2021) 67:290–7. doi: 10.1177/0020764020950775 32815441

[19] Kuper LE Stewart S Preston S Lau M Lopez X. Body dissatisfaction and mental health outcomes of youth on gender-affirming hormone therapy. Pediatrics. (2020) 145:e20193006. doi: 10.1542/peds.2019-3006 32220906

[20] Biggs M. Suicide by clinic-referred transgender adolescents in the United Kingdom. Arch Sex Behav. (2022) 51:685–90. doi: 10.1007/s10508-022-02287-7 35043256 PMC8888486

[21] Tordoff DM Wanta JW Collin A Stepney C Inwards-Breland DJ Ahrens K. Mental health outcomes in transgender and nonbinary youths receiving gender-affirming care. JAMA Netw Open. (2022) 5:e220978. doi: 10.1001/jamanetworkopen.2022.0978 35212746 PMC8881768

[22] Foster Skewis L Bretherton I Leemaqz SY Zajac JD Cheung AS. Short-term effects of gender-affirming hormone therapy on dysphoria and quality of life in transgender individuals: a prospective controlled study. Front Endocrinol. (2021) 12:717766. doi: 10.3389/fendo.2021.717766 34394009 PMC8358932

[23] Costa R Dunsford M Skagerberg E Holt V Carmichael P Colizzi M. Psychological support, puberty suppression, and psychosocial functioning in adolescents with Gender Dysphoria. J Sex Med. (2015) 12:2206–14. doi: 10.1111/jsm.13034 26556015

[24] Reisner SL Vetters R Leclerc M Zaslow S Wolfrum S Shumer D . Mental health of transgender youth in care at an adolescent urban community health center: a matched retrospective cohort study. J Adolesc Health. (2015) 56:274–9. doi: 10.1016/j.jadohealth.2014.10.264 25577670 PMC4339405

[25] Toomey RB Syvertsen AK Shramko M. Transgender adolescent suicide behavior. Pediatrics. (2018) 142:e20174218. doi: 10.1542/peds.2017-4218 30206149 PMC6317573

[26] Majumder A Roychaudhuri S Chakraborty S Bhattacherjee K. An observational study of the quality of life among gender incongruent individuals from “Hijra” community of India. Indian J Endocrinol Metab. (2020) 24:301–5. doi: 10.4103/ijem.IJEM_169_20 33088751 PMC7540832

[27] Sevelius JM. Gender Affirmation: A framework for conceptualizing risk behavior among transgender women of color. Sex Roles. (2013) 68:675–89. doi: 10.1007/s11199-012-0216-5 23729971 PMC3667985

[28] Dhejne C Lichtenstein P Boman M Johansson AL Långström N Landén M. Long-term follow-up of transsexual persons undergoing sex reassignment surgery: cohort study in Sweden. PLoS ONE. (2011) 6:e16885. doi: 10.1371/journal.pone.0016885 21364939 PMC3043071

[29] Lindqvist EK Sigurjonsson H Möllermark C Rinder J Farnebo F Lundgren TK. Quality of life improves early after gender reassignment surgery in transgender women. Eur J Plast Surg. (2017) 40:223–6. doi: 10.1007/s00238-016-1252-0 28603386 PMC5440516

[30] Olson-Kennedy J Streeter LH Garofalo R Chan YM Rosenthal SM. Histrelin Implants for Suppression of Puberty in Youth with Gender Dysphoria: A Comparison of 50 mcg/Day (Vantas) and 65 mcg/Day (SupprelinLA). Transgend Health. (2021) 6:36–42. doi: 10.1089/trgh.2020.0055 33644320 PMC7906230

[31] Chen D Berona J Chan YM Ehrensaft D Garofalo R Hidalgo MA . Psychosocial functioning in transgender youth after 2 years of hormones. N Engl J Med. (2023) 388:240–50. doi: 10.1056/NEJMoa2206297 36652355 PMC10081536

[32] Hatzenbuehler ML Pachankis JE. Stigma and minority stress as social determinants of health among lesbian, gay, bisexual, and transgender youth: research evidence and clinical implications. Pediatr Clin North Am. (2016) 63:985–97. doi: 10.1016/j.pcl.2016.07.003 27865340

[33] Azagba S de Silva GSR. Ebling T. Examining general, physical, and mental health disparities between transgender and cisgender adults in the US. Int J Equity Health. (2025) 24:37. doi: 10.1186/s12939-024-02364-4 39905458 PMC11796085

[34] Bradford J Reisner SL Honnold JA Xavier J. Experiences of transgender-related discrimination and implications for health: results from the Virginia Transgender Health Initiative Study. Am J Public Health. (2013) 103:1820–9. doi: 10.2105/AJPH.2012.300796 23153142 PMC3780721

[35] de Graaf NM Cohen-Kettenis PT Carmichael P de Vries ALC Dhondt K Laridaen J . Psychological functioning in adolescents referred to specialist gender identity clinics across Europe: a clinical comparison study between four clinics. Eur Child Adolesc Psychiatry. (2018) 27:909–19. doi: 10.1007/s00787-017-1098-4 29256158 PMC6013511

[36] Bhattacharya S Ghosh D Purkayastha B. ‘Transgender persons (protection of rights) act' of india: an analysis of substantive access to rights of a transgender community. J Hum Rights Pract. (2022) 14:676–97. doi: 10.1093/jhuman/huac004 36246149 PMC9555747

[37] Dutta A. An epistemology of collusion: Hijras, Kothis and the historical (dis)continuity of gender/sexual identities in Eastern India. Gender Hist. (2012) 24:825–849. doi: 10.1111/j.1468-0424.2012.01712.x

[38] Craig SL Eaton AD Leung VWY Iacono G Pang N Dillon F . Efficacy of affirmative cognitive behavioural group therapy for sexual and gender minority adolescents and young adults in community settings in Ontario, Canada. BMC Psychol. (2021) 9:94. doi: 10.1186/s40359-021-00595-6 34099063 PMC8183324

[39] Kosciw JG Greytak EA Bartkiewicz MJ Boesen MJ Palmer NA. The 2011 National School Climate Survey: the experiences of lesbian, gay, bisexual and transgender youth in our nation's schools. New York, NY: GLSEN (2012).

[40] Pachankis JE Soulliard ZA Layland EK Behari K Seager van Dyk I Eisenstadt BE . Guided LGBTQ-affirmative internet cognitive-behavioral therapy for sexual minority youth's mental health: a randomized controlled trial of a minority stress treatment approach. Behav Res Ther. (2023) 169:104403. doi: 10.1016/j.brat.2023.104403 37716019 PMC10601985

