# Supplementary material for: Psychosocial and mental health outcomes associated with gender-affirming interventions among transgender youth: a scoping review
Source: Front Public Health. 2026 Sep 16;14:1910466. doi: 10.3389/fpubh.2026.1910466 (PMC13625275; doi:10.3389/fpubh.2026.1910466)
Supplement: Supplementary file 1 [file Supplementary_file_1.docx]

**Supplementary material 1.** **Characteristics and Key Findings of Included Studies on Gender-Affirming Interventions and Psychosocial Outcomes among Transgender Youth**

| **First author, year** | **Country** | **Study**  **design** | **Sample**  **size** | **Study population** | **Type of gender-affirming intervention** | **Stage/**  **Duration** | **Outcomes assessed** | **Measurement tools** | **Key findings** | **Conclusion** |
| --- | --- | --- | --- | --- | --- | --- | --- | --- | --- | --- |
| Strauss P et al. (2020)^9^ | Australia | Cross-sectional | n=859 | Trans and gender diverse youth; N = 859; ages 14-25; identities included trans male, trans female, nonbinary, genderqueer, agender | Not intervention-focused; reports social transition, peer support, and mental-health service engagement (no medical intervention measured) | Not applicable | Depression, anxiety, self-harm, suicidality, psychological distress, impact of negative life events, access to services | Kessler Psychological Distress Scale; suicidality/self-harm items; negative life events checklist | Extremely high distress (Kessler Psychological Distress Scale); 79% self-harmed, 48% attempted suicide; family rejection, homelessness, and discrimination predicted distress; supportive healthcare interactions reduced risk | TGD youth face severe mental-health burdens due to trauma and systemic discrimination; access to affirming mental-health services and supportive environments is essential |
| Pelusi C et al., (2014)^15^ | Italy | Prospective cohort | n=45 | FTM transgender persons; N = 45 FTM individuals | GAHT - three different testosterone formulations (intramuscular depot, testosterone gel, testosterone undecanoate) | 12 months (baseline, week 30, week 54) | Body composition (lean and fat mass), metabolic and biochemical parameters, bone / hematological, life satisfaction | Anthropometry, body composition measures, lipid profile, coagulation panel, amenorrhea status, self-reported life satisfaction | After 1 year of testosterone therapy: lean mass ↑, fat mass ↓, HDL ↓, LDL ↑, prothrombin time ↑; all subjects became amenorrheic; general life satisfaction increased in all groups | One-year testosterone treatment appears broadly safe across formulations, produces virilization and body composition changes, and increases life satisfaction in FTM transgender persons |
| Tucker RP et al., (2018)^16^ | USA | Cross-sectional | n=155 | Transgender N = 155; included transgender | Hormone therapy (estradiol/testosterone) and gender-affirming surgeries (top/bottom/facial); mental-health counseling and peer support available | Cross-sectional analysis; participants included pre-treatment, currently on HRT, and post-surgical veterans | Suicidal ideation, suicide attempts, depression, psychological distress | Columbia Suicide Severity Rating Scale; depression/distress self-report measures | Veterans who had undergone gender-affirming surgery showed significantly lower suicidality; hormone therapy users also reported fewer suicide attempts; untreated dysphoria strongly predicted self-harm | Gender-affirming surgeries and hormone therapy are associated with lower suicide risk among transgender veterans; mental-health support remains essential |
| Poguri M et al., (2016)^17^ | India | Cross- sectional | n=15 | Fifteen transfeminine individuals median age 30 years | Observational pilot study (does not evaluate HRT/blockers/surgery outcomes) | Not applicable | Emotional distress, anxiety, depression, general well-being, QoL domains, suicide behavior, trauma | Hospital Anxiety and Depression Scale, General Health Questionnaire-12, World Health Organization Quality of Life-BREF | 100% reported childhood sexual abuse; 73.3% had lifetime suicide attempts; 66.7% had significant anxiety, 100% had depressive symptoms; Social QoL domain extremely low | Transgender individuals in South India experience very high psychological distress and poor social QoL; healthcare providers must adopt sensitive, inclusive care approaches |
| Sartaj D et al., (2021)^18^ | India | Cross-sectional | n=50 | Hijra/Transgender adults living in community settings across urban/semi-urban regions; sample comprises transfeminine Hijra identity | Social gender transition through adoption of Hijra identity; no medical interventions reported | Not applicable (non-intervention study) | Mental health, depression, anxiety, self-esteem, QoL, stigma impacts | Standardized psychological scales (names unspecified in snippet) | Elevated depression/anxiety; low self-esteem strongly linked to discrimination; stigma predicted poorer outcomes; Hijra community networks provided emotional support | Hijra individuals face severe psychological burden due to stigma, exclusion, and economic vulnerability; community support acts as a partial protective factor, indicating need for structural and mental-health interventions |
| Kuper LE et al., (2020)^19^ | USA | Prospective cohort | n=30 | Transgender and nonbinary adolescents (N = 30; ages 13–17) | Affirming cognitive behavioral therapy | 12-week Cognitive behavioral therapy intervention | Depression, anxiety, psychological well-being | Standardized mental-health scales Beck Depression Inventory, anxiety measures | Significant reduction in depressive symptoms and improved well-being after affirming cognitive behavioral therapy program | Gender-affirming psychotherapy is effective and improves adolescent mental health |
| Biggs M (2020)^20^ | UK | Retrospective | n=44 | Adolescents at UK Gender Identity Development Service; N = 44 | Puberty blockers (GnRH agonists) | Follow-up: 12–24 months after treatment start | Psychological functioning, self-harm, bone density | Child Behavior Checklist, Youth Self Report, clinical records | No statistically significant improvement in psychological functioning; some increase in self-harm reported in subgroup | Findings limited by small sample and lack of control group; results do not support detransition but call for cautious evaluation of outcomes |
| Tordoff DM et al., (2022)^21^ | USA | Prospective cohort | n=104 | Transgender and nonbinary adolescents (ages 13–20) attending Seattle Children’s Gender Clinic; N = 104 initiating puberty blockers or HRT | Puberty blockers (GnRH agonists) and hormone therapy (testosterone/estrogen); psychosocial counseling available; family support encouraged | 3-, 6-, and 12-month follow-up after initiating medical care | Depression, anxiety, suicidal ideation/self-harm, overall psychological well-being | Patient Health Questionnaire (depression),Generalized Anxiety Disorder–7 (anxiety), suicidality/self-harm subscales | Sixty-percent reduced odds of moderate–severe depression (adjusted odds ratio 0.40) and 73% reduced odds of suicidality (adjusted odds ratio 0.27) at 12 months; anxiety improved by 6 months; greatest reductions seen in youth starting both blockers and HRT | Gender-affirming medical care significantly improves mental health and reduces depression and suicidality within one year; early access is beneficial |
| Foster Skewis L et al., (2021)^22^ | Australia | Prospective cohort | n=180 | Initiating GAHT; N = 77 trans (masculinizing n=42; feminizing n=35) and + 103 cisgender controls | GAHT (testosterone or estradiol); no psychosocial treatment measured | Prospective follow-up after starting GAHT (baseline → 3 → 6 months) | QoL, gender dysphoria, emotional well-being, social functioning | Gender Preoccupation and Stability Questionnaire; RAND Short Form Health Survey-36 | Masculinizing GAHT: ↓ dysphoria; ↑ emotional well-being and social functioning. Feminizing GAHT: ↓ dysphoria; QoL effects less pronounced in short term | GAHT reduces dysphoria; masculinizing therapy also yields early improvements in psychosocial QoL domains; feminizing effects may require longer follow-up or additional support |
| Costa R et al., (2015)^23^ | UK | Prospective cohort | n=201 | Adolescents diagnosed with gender dysphoria; N = 201 at baseline. | Psychological support for all; subgroup also received puberty suppression (GnRHa) | Longitudinal follow-up assessments every 6 months; 12-month follow-up for suppression subgroup (and up to 18 mo total follow-up per some reports) | Global psychosocial functioning; gender-dysphoria related distress | (CGAS) for functioning; Utrecht Gender Dysphoria Scale for gender dysphoria discomfort. | - Baseline CGAS: 57.7 ± 12.3 (indicating poor functioning) - After 6 months of psychological support (no GnRHa): CGAS improved to 60.7 ± 12.5 (p < 0.001) - After 12 months with puberty suppression and support: subgroup receiving GnRHa had CGAS = 67.4 ± 13.9, significantly higher compared with the support-only group (CGAS = 60.9 ± 12.2; p = 0.001) | Psychological support alone led to modest improvement in global functioning; puberty suppression (with ongoing support) associated with greater functional improvement than support alone |
| Reisner et al., (2015)^24^ | USA | Cross-sectional | n=452 | Transgender youths N = 452; diverse gender identities | None (observational; focused on disparities, not treatment) | Not applicable | Depression, suicidality, discrimination, barriers to care | Standardized mental-health questionnaires and survey measures | High rates of depression and suicidality strongly linked to discrimination and lack of access to gender-affirming healthcare | Reducing discrimination and improving access to gender-affirming services are critical to improve mental health |
| Toomey RB et al., (2018)^25^ | USA | Cross-sectional | n=120,617 | National sample of adolescents aged 11–19; N = 120,617 | None (observational prevalence study; no medical or psychosocial intervention measured) | Not applicable | Lifetime suicide attempts (primary); demographic and psychosocial correlates across gender identity subgroups | Self-reported survey item on suicide attempts; demographic self-report; no psychological scales | Suicide attempts were highest among FTM (50.8%), nonbinary youth (41.8%), MTF (29.9%), compared to cisgender girls (17.6%) and cisgender boys (9.8%); sexual minority identity further increased risk; demographic factors showed little protection | Suicide risk varies sharply across gender identities; transgender and nonbinary youth—especially FTM—require targeted prevention, affirming environments, and support due to extreme vulnerability |
| Majumder A et al., (2020)^26^ | India | Cross-sectional | n=74 | Hijra group: 37 participants  Control group: 37 participants | Observational (study does not test any gender-affirming medical/surgical intervention) | Not applicable | QoL, depression, anxiety, minority stress experiences | World Health Organization Quality of Life-BREF, Patient Health Questionnaire–9, Generalized Anxiety Disorder-7, Minority Stress assessment parameters | Social and environmental domains of QoL were lowest; 74% had moderate–severe depression; 66% had moderate–severe anxiety; QoL positively correlated with family/social support; discrimination strongly predicted poorer well-being | Mental-health burden is high in Indian transgender populations; improving social support, family acceptance and healthcare accessibility is essential |
| Sevelius JM (2013)^27^ | USA | Qualitative study (secondary analysis of two qualitative studies) | n=22 | Twenty-two transgender women of color from the San Francisco Bay Area; ages 20–65 years | Gender affirmation as a psychosocial framework (not a tested medical intervention); mentions risk behaviors including unsafe body modification practices | Not a clinical trial | Unmet need for gender affirmation as a driver for HIV risk, psychological distress, and coping behaviors | Qualitative semi-structured interviews; intersectional analysis grounded in stigma and identity threat models | Risk behavior including unsafe body modification and sexual risk is conceptualized as a response to unmet gender affirmation needs in the context of racism, sexism and transphobia | Gender-affirmation needs directly influence health behavior; interventions that increase gender affirmation may reduce high-risk health behaviors among transgender women of color |
| Dhejne C et al., (2011)^28^ | Sweden | Retrospective | n=324 | Transgender (N = 324) who completed gender affirmation (legal and surgical); population-based cohort using national registers | Gender-affirming surgery and GAHT (standard for cohort); no analysis of psychosocial interventions | Long-term follow-up post-surgery (up to 30 years; data 1973–2003) | Mortality, suicide deaths and attempts, psychiatric inpatient care, criminal convictions | National registers: Cause of Death, Hospital Discharge (psychiatry), Crime Register, Total Population Register | Higher mortality vs controls (adjusted Hazard Ratio 2.8); markedly elevated suicide deaths (adjusted Hazard Ratio 19.1) and attempts (adjusted Hazard Ratio 4.9); increased psychiatric hospitalizations (adjusted Hazard Ratio 2.8) | Medical transition reduces dysphoria but does not eliminate long-term psychiatric risks; sustained mental-health and somatic follow-up are essential |
| Lindqvist EK et al., (2017)^29^ | Sweden | Prospective cohort | n=190 | Transgender women undergoing gender affirming surgery; N = 190; age 19–76 (mean 36) | Gender affirming surgery | Prospective follow-up pre-op → 1, 3, 5 years post-op | QoL (8 domains), general perceived health | Swedish Short Form Health Survey–36 | QoL lower at baseline vs general population; significant improvement at 1 year; slight non significant decline at 3–5 years; perceived health peaks at 1 year | Early surgical transition improves QoL; long-term levels may reflect aging/post-surgical expectations; ongoing follow-up recommended |
| Olson-Kennedy J et al., (2021)^30^ | USA | Prospective cohort | n=66 | Sixty-six transgender youth in early–mid puberty (Tanner stage 2–3) receiving puberty blockers | Puberty suppression using Histrelin implant – Vantas (50 mcg/day) vs SupprelinLA (65 mcg/day) | 2–12 months follow-up | Suppression of gonadotropins (LH, FSH), suppression of sex steroids (testosterone, estradiol) | Serum LH, FSH, estradiol, testosterone levels | Both Vantas and Supprelin LA produced equivalent suppression of the hypothalamic pituitary-gonadal axis. Significant decrease in LH, FSH, testosterone/estradiol from baseline. No meaningful clinical differences between the two implants. | Both implants are effective for puberty suppression. Cheaper Vantas implant performs as well as Supprelin LA, supporting its use as a cost-effective option in transgender youth care. |

CGAS, Children’s Global Assessment Scale; FSH, follicle-stimulating hormone; FTM, female-to-male; GAHT, gender-affirming hormone therapy; GnRH, gonadotropin-releasing hormone; GnRHa, gonadotropin-releasing hormone agonist; HDL, high-density lipoprotein; HRT, hormone replacement therapy; LDL, low-density lipoprotein; LH, luteinizing hormone; MTF, male-to-female; QoL, quality of life; TGD, transgender and gender diverse.
